# Multi-omics approaches for image classification in disease diagnosis

**DOI:** 10.3389/fcimb.2025.1616189

**Published:** 2025-12-04

**Authors:** Yan Lin, Shu Chen, Jinshan Che, Mingming Sun, Yuhong Wang

**Affiliations:** 1 Department of Critical Care Medicine, Clinical Oncology School of Fujian Medical University, Fujian Cancer Hospital, NHC Key Laboratory of Cancer Metabolism, Fuzhou, China; 2 Department of Gastric Surgery, Clinical Oncology School of Fujian Medical University, Fujian Cancer Hospital, NHC Key Laboratory of Cancer Metabolism, Fuzhou, China; 3 Fourth Clinical College of Xinxiang Medical College, Xinxiang Central Hospital, Xinxiang, Henan, China

**Keywords:** multi-omics integration, disease microbiology, image classification, host-pathogen dynamics, equilibrium inference strategy

## Abstract

**Introduction:**

The integration of multi-omics data for disease diagnosis holds transformative potential in the field of computational biology, especially when applied to the intricate and dynamic interactions between microbial communities and their human hosts.

**Methods:**

This integrative approach enables to capture diverse biological signals across genomic, transcriptomic, proteomic, and metabolomic layers, providing a more comprehensive understanding of disease mechanisms. In alignment with emerging priorities in disease microbiology, our study addresses a critical and timely need for interpretable, scalable, and biologically robust computational models that can extract clinically meaningful diagnostic insights from inherently high-dimensional, heterogeneous, and often incomplete biological datasets.

**Results and Discussion:**

Traditional image classification approaches in disease contexts—such as those relying solely on histopathological features or genomic imaging—tend to overlook the broader ecological and systemic dimensions that are essential for decoding the mechanisms of microbial pathogenesis. These single-modal methods often suffer from significant limitations, including reduced scalability to diverse clinical settings, poor generalizability across patient populations, and an inability to handle partially observed or biologically variable data. Such constraints diminish their effectiveness in precision diagnostics, disease subtyping, and therapeutic decision-making. By contrast, our approach emphasizes multi-modal integration and model interpretability, aiming to overcome these limitations and advance the development of next-generation diagnostic tools that are both clinically actionable and biologically grounded.

## 1 Introduction

The integration of multi-omics data with imaging has emerged as a promising direction for disease diagnosis, driven by the need for more accurate, early, and individualized medical assessments (Chen et al., 2021a). Traditional imaging alone often lacks the molecular-level specificity necessary to capture early or subtle pathological changes (Maurício et al., 2023). Conversely, omics data—such as genomics, transcriptomics, proteomics, and metabolomics—offer comprehensive biological insights but lack spatial context. Not only does a multi-omics approach provide a more holistic view of disease mechanisms, but it also enhances the interpretability of image-based models through biologically relevant features (Touvron et al., 2021). Furthermore, fusing omics layers with imaging data enables more robust and generalizable classification systems, particularly critical for complex, multifactorial diseases like cancer and neurodegenerative disorders (Hong et al., 2021). This convergence supports personalized medicine, as it can tailor diagnosis and treatment strategies to individual patients based on a combination of molecular signatures and visual pathology, thus redefining the landscape of disease classification and prognosis (Tian et al., 2020).

In the initial phase of development, researchers attempted to link biological and imaging information by formulating expert-driven frameworks that relied on structured associations between annotated image characteristics and known disease features (Hong et al., 2020). These systems, often built on domain knowledge and manually encoded rules, offered a degree of interpretability and control over diagnostic logic, making them attractive in controlled or narrowly defined clinical contexts (Yang et al., 2021b). Typically, these frameworks function by aligning predefined imaging phenotypes with curated biological markers, facilitating early attempts at multi-modal integration. However, their performance deteriorated in the face of real-world complexity, where biological data are incomplete, noisy, and not easily described by fixed patterns (Sun et al., 2022). The brittle nature of rule-based models, coupled with their reliance on comprehensive annotation and expert supervision, made them poorly suited for dynamic or large-scale datasets (Rao et al., 2021). Moreover, the lack of scalability and adaptability significantly limited their applicability to broader clinical settings, especially when integrating multi-omics layers with heterogeneous structures and missing values was required (Mai et al., 2021).

To move beyond these limitations, a second wave of approaches employed statistical models and algorithmic pipelines that could learn from examples rather than fixed rules (Wang et al., 2022). These methods established more flexible paradigms by combining numerical features derived from omics profiles with visual traits extracted from medical images, allowing for semi-automated analysis and classification (Azizi et al., 2021). By introducing statistical learning principles into diagnostic pipelines, these approaches facilitated pattern recognition across diverse patient populations and provided a foundation for multi-modal fusion (Li et al., 2020). While this led to measurable gains in predictive power and opened the door to more robust multi-modal analysis, their effectiveness was often constrained by the need for meticulous feature engineering, reliance on domain-specific preprocessing pipelines, and susceptibility to overfitting in high-dimensional settings (Bhojanapalli et al., 2021). The rigid separation between imaging and omics modalities, combined with challenges in modeling their interactions, hindered the discovery of complex cross-modal relationships necessary for deeper biological insight and clinically meaningful interpretation (Kim et al., 2022).

In recent years, advances in computational frameworks have ushered in a new generation of integrative models capable of learning hierarchical, cross-modal representations directly from raw, high-dimensional data (Zhang et al., 2020). By leveraging neural architectures adept at capturing both spatial and contextual information, including convolutional networks, attention mechanisms, and graph-based embeddings, these models facilitate end-to-end learning pipelines that unify imaging and omics data at multiple abstraction levels (Roy et al., 2022). Such integration allows for the discovery of subtle, non-obvious patterns that are critical for accurate disease classification, outcome prediction, and biomarker identification (Zhu et al., 2020). Importantly, these models have shown the ability to generalize across diverse cohorts, adapt to missing or partially observed data, and highlight biologically meaningful interactions between cellular structure and molecular function (Chen et al., 2021b). Despite persistent challenges related to harmonizing data resolution, modality-specific noise, model transparency, and computational resource demands, these integrative systems have demonstrated superior performance and adaptability in diagnosing multifactorial diseases. They mark a significant step toward the realization of truly personalized, multi-modal, and data-integrated medical decision-making in the era of precision health (Ashtiani et al., 2021).

Based on the limitations of symbolic reasoning, the reliance on handcrafted features in machine learning, and the integration challenges in deep learning, we propose a novel framework that unifies imaging and multi-omics data through a cross-modal attention mechanism and dynamic graph fusion. This approach is designed to address both the heterogeneity and the complementarity of imaging and omics modalities. By aligning visual and molecular information within a shared representational space, our method not only facilitates deeper biological interpretation but also improves model generalizability. Moreover, the proposed system incorporates domain-specific priors to enhance interpretability while retaining the adaptability of end-to-end learning. The motivation behind our method stems from the need to create a cohesive pipeline that overcomes existing bottlenecks in feature alignment, modality fusion, and diagnostic reliability, particularly in real-world, heterogeneous patient datasets. Our framework is designed to integrate comprehensive biological understanding with advanced visual analytics, thereby advancing the field of precision diagnostics.

- The proposed method introduces a novel cross-modal attention module tailored for aligning multi-omics data with spatial imaging features, enabling more effective fusion of heterogeneous data.
- It features a dynamic graph-based fusion mechanism that adapts to varying data structures, supporting multi-disease scenarios, enhancing computational efficiency, and offering strong generalizability.
- Experimental results on benchmark disease datasets demonstrate superior classification accuracy, robustness across modalities, and improved interpretability compared to existing state-of-the-art methods.

## 2 Related work

### 2.1 Integration of multi-omics data

The integration of multi-omics data has emerged as a powerful strategy to enhance disease diagnosis by capturing the biological complexity underlying disease phenotypes (Masana et al., 2020). Multi-omics data typically include genomics, transcriptomics, proteomics, metabolomics, and epigenomics. Each omics layer offers distinct but complementary information about the molecular state of a biological system. In disease diagnosis, especially for complex diseases such as cancer and neurodegenerative disorders, single-omics approaches often fail to capture the full spectrum of molecular alterations. Integrative approaches aim to combine these heterogeneous data types to achieve more comprehensive insights into disease mechanisms and more accurate predictions (Lahiri et al., 2019). Data integration strategies can be broadly classified into early integration (concatenation-based), intermediate integration (model-based), and late integration (decision-based) methods. Early integration methods simply concatenate features from each omics layer into a single feature vector, which is then used to train machine learning models. While straightforward, this approach suffers from the curse of dimensionality and potential information redundancy (Zhang et al., 2022). Intermediate integration strategies use algorithms such as canonical correlation analysis, kernel methods, or deep learning to find shared representations between omics datasets before classification. Late integration methods involve training separate models for each omics layer and then combining their outputs through ensemble methods or voting schemes (Lahiri et al., 2006). Recent advancements in deep learning have further enabled the development of more sophisticated intermediate integration strategies. For instance, autoencoders and variational autoencoders have been used to learn compact, non-linear embeddings of multi-omics data, which can then be fused with image features for downstream classification. Multi-modal deep learning frameworks that simultaneously process omics and image data streams have demonstrated improved performance in disease classification tasks (Mascarenhas and Agarwal, 2021). In the context of image classification, multi-omics data often serve as complementary inputs to imaging features extracted from modalities such as MRI, CT, or histopathology (Roy et al., 2023a). For example, in cancer diagnosis, integrating gene expression data with histopathological images has been shown to improve the prediction of tumor subtypes and patient outcomes. The integration enhances the interpretability of the image features by linking them to molecular pathways and biological processes. The primary challenges in multi-omics integration include data heterogeneity, missing values, and the need for large, annotated datasets. Furthermore, effective feature selection and normalization techniques are critical to mitigate the effects of noise and batch effects. Addressing these challenges requires a combination of domain knowledge, computational innovation, and rigorous validation protocols (He et al., 2025).

In recent years, a number of computational tools have been developed to address the challenge of multi-omics data integration in disease analysis and patient stratification. Methods such as PINSPlus (Nguyen et al., 2019) and Cancer Integration via Multikernel Learning (CIMLR) (Ramazzotti et al., 2018) have demonstrated the utility of combining genomic, transcriptomic, and epigenomic profiles to discover tumor subtypes, using consensus clustering and similarity-based fusion, respectively. More advanced frameworks like iCluF (Shakyawar et al., 2024) employ iterative cluster-fusion strategies to enhance stability in unsupervised multi-omics integration, while Subtype-GAN (Yang et al., 2021a) applies generative adversarial networks to improve latent feature extraction across heterogeneous biological data sources. These tools have shown that integrating molecular layers leads to more biologically meaningful subtyping and better clinical interpretability (Kautish et al., 2021). Beyond cancer subtyping, integration models have also been extended to public health and epidemiological contexts. For example, Shakyawar et al. (2021) proposed a big data framework that jointly models multi-omics data and comorbidities in the context of COVID-19 and systemic diseases, demonstrating the feasibility of applying integrative analytics beyond oncology. These prior efforts underscore the methodological relevance of multi-modal fusion in complex disease modeling. Building upon this foundation, our study introduces a visual–omics framework that complements these integration strategies by embedding visual pathology data into the multi-omics learning process. Compared to existing methods, our approach incorporates spatial, immune, and ecological dynamics, offering a unified probabilistic representation suitable for both classification and latent inference under real-world biological constraints.

### 2.2 Deep learning in medical imaging

Deep learning has revolutionized the field of medical imaging by enabling the automated extraction of hierarchical features from complex image data. Convolutional neural networks (CNNs), in particular, have demonstrated remarkable success in tasks such as image classification, segmentation, and detection across various imaging modalities, including MRI, CT, ultrasound, and digital pathology (Woo et al., 2023a). The use of deep learning in disease diagnosis has become prevalent due to its ability to learn discriminative features that may not be visible to the human eye (Taori et al., 2020). Traditional machine learning approaches relied heavily on handcrafted features, which are not only labor-intensive to design but also limited in their ability to capture high-level semantic information. Deep learning models, however, learn feature representations directly from raw image pixels in a data-driven manner. This capability has led to significant improvements in diagnostic accuracy, especially in the classification of diseases such as diabetic retinopathy, lung cancer, Alzheimer’s disease, and breast cancer (Peng et al., 2022). In multi-omics-based image classification, deep learning frameworks can be used to fuse visual and molecular data, leading to more robust and biologically meaningful predictions. Several architectures have been proposed for this purpose, including multi-branch networks where separate CNN branches process image and omics data before combining them in a shared representation space (Lahiri et al., 2012). Attention mechanisms and graph neural networks have also been utilized to enhance the model’s ability to focus on relevant features across different data modalities. Transfer learning and pretrained models play a crucial role in medical imaging, particularly when labeled data are scarce. Models trained on large datasets such as DIBaS can be fine-tuned on medical imaging tasks to achieve better generalization. Data augmentation techniques and synthetic image generation using generative adversarial networks (GANs) have also been employed to overcome data limitations (Bazi et al., 2021). Despite these advancements, challenges remain in terms of model interpretability, generalization across institutions, and regulatory approval for clinical deployment. Explainable Artificial Intelligence (AI) techniques, such as saliency maps and class activation maps, are increasingly being integrated into deep learning workflows to provide insights into model decisions and enhance clinical trust (Lu et al., 2025).

In recent years, attention mechanisms have emerged as a transformative component in deep learning models, particularly in the field of medical imaging. Unlike traditional convolutional operations that rely on fixed receptive fields, attention modules dynamically weigh the importance of spatial, channel-wise, or cross-modal features, enabling models to focus on the most informative regions of the input data. This has led to significant improvements in tasks such as lesion localization, fine-grained classification, and modality fusion. For instance, self-attention modules in transformer-based architectures, such as Vision Transformers (ViT), have demonstrated competitive performance in radiology and pathology tasks by capturing long-range dependencies and contextual interactions more effectively than CNNs. Cross-attention designs have been employed to link image features with complementary data modalities, such as gene expression profiles or clinical metadata, supporting more robust diagnosis under heterogeneous data conditions. Recent works have also explored the integration of attention into multi-branch architectures, where image and omics features are processed separately and then aligned via learnable attention weights. These strategies improve the interpretability and adaptability of medical AI models, especially in scenarios with partially missing or noisy modalities. In our work, attention is used as a core mechanism in both spatial feature refinement and modality-level fusion. Specifically, we adopt a cross-modal attention layer that aligns omics-derived embeddings with spatial image features, allowing the model to selectively integrate molecular signals in a region-aware manner. This design choice is supported by the growing body of literature that demonstrates the superiority of attention-based fusion in medical image understanding and multi-modal learning.

### 2.3 Multi-modal fusion techniques

Multi-modal fusion refers to the computational techniques used to integrate data from different modalities to improve the performance and reliability of disease diagnosis models. In the context of multi-omics and image data, fusion techniques are critical for leveraging the complementary nature of visual and molecular information. Effective fusion not only enhances classification performance but also improves biological interpretability and clinical relevance (Bugatti et al., 2025a). Fusion techniques are generally categorized into three levels, including data, feature, and decision levels. Data-level fusion combines raw data from different sources before any processing, which is rare due to the differences in data structure and dimensionality (Zhang et al., 2025a). Feature-level fusion extracts features from each modality separately and then concatenates or combines them using methods such as tensor fusion, bilinear pooling, or attention-based mechanisms. Decision-level fusion aggregates the outputs of separate classifiers, using ensemble methods such as majority voting or stacking (Zheng et al., 2022). Advanced feature-level fusion methods have been developed using deep learning. These include architectures like cross-modal transformers, dual-stream CNNs, and hybrid networks that incorporate recurrent layers or attention modules. Such models are capable of capturing complex relationships between modalities and can dynamically weigh the importance of each modality during classification. Multi-modal fusion has been applied successfully in several disease diagnosis tasks (Bugatti et al., 2025a). For instance, in glioma classification, combining MRI features with gene expression profiles has led to more accurate predictions of tumor grade and molecular subtype. Similarly, integrating histopathology images with proteomic and transcriptomic data has enhanced the identification of prognostic biomarkers in breast cancer (Dai and Gao, 2021). An important aspect of multi-modal fusion is the alignment of data across modalities, both temporally and spatially. For example, aligning biopsy samples with corresponding imaging data requires careful annotation and registration techniques. Moreover, data normalization and dimensionality reduction are essential preprocessing steps to ensure that the fused features are compatible and informative (Zhang et al., 2025a). The success of multi-modal fusion depends on the availability of high-quality, well-annotated datasets, as well as on computational frameworks that can efficiently handle the scale and complexity of multi-omics and imaging data. With continued advancements in data acquisition technologies and computational methods, multi-modal fusion is poised to play a central role in the next generation of precision medicine tools (Dong et al., 2022).

## 3 Method

### 3.1 Overview

Disease microbiology represents a pivotal field that investigates the interactions between microbial agents and their hosts, focusing on the molecular, cellular, and ecological aspects of infectious diseases. This subsection aims to provide a comprehensive introduction to the methodological framework and conceptual underpinnings that form the basis of our proposed approach. We outline the structure and contributions of the ensuing sections, highlighting their roles in the broader methodology.

We begin by formally defining the core research problem and mathematical structure in Section 3.2. This part introduces a symbolized abstraction of the disease microbiology setting, capturing both host–pathogen dynamics and the multi-scale interactions that influence disease progression. The section lays out the notational foundation, integrating principles from epidemiology, molecular biology, and systems microbiology. The mathematical formulations not only enable a deeper understanding of pathogen behaviors under various environmental and immunological contexts but also provide a tractable basis for model development and analytical reasoning. Following the foundational formalism, Section 3.3 introduces a biologically inspired computational architecture tailored for disease microbiology. The new model encapsulates pathogen evolution, host responses, and microenvironmental feedback using a dynamic, structured representation. In contrast to conventional mechanistic or statistical models, our design leverages advances in probabilistic graphical modeling and latent variable learning to simulate the heterogeneous trajectories of infection. It incorporates high-dimensional, multi-modal datasets and is engineered to accommodate partial observations and inherent biological variability. Moreover, it addresses challenges in modeling latent reservoirs, horizontal gene transfer, and cross-species spillover with novel algorithmic components. Subsequently, in Section 3.4, we detail a bespoke inference strategy that facilitates the efficient training and deployment of the proposed model. Recognizing the complexity of microbial interaction networks, this strategy employs an adaptive, energy-based exploration mechanism to identify equilibrium states in host–pathogen systems. It integrates domain-specific priors, such as host susceptibility maps and known resistance loci, to guide search trajectories and optimize model fidelity. The strategy is designed to handle large-scale data inputs, including metagenomic sequencing, gene expression profiles, and spatial–temporal disease incidence patterns. It further incorporates mechanisms to balance accuracy and generalizability, enabling robust performance across diverse biological systems and disease contexts.

### 3.2 Preliminaries

In this section, we formalize the problem of disease microbiology from a symbolic and mathematical perspective, focusing on the intrinsic dynamics between pathogens, host immune responses, and their surrounding environment. The goal is to construct an abstracted, mathematically tractable representation that captures the complexity of microbial infections and host–pathogen interactions over time and space. This formulation provides the foundation upon which our modeling and inference methods are built.

Let P denote the set of all microbial pathogens under consideration, indexed by p∈P. Let ℋ denote the set of hosts, indexed by h∈ℋ. Each pathogen p is associated with a genomic signature $g_{p}\in {\mathbb{R}}^{d_{g}}$ and a set of virulence factors $v_{p}\in {\mathbb{R}}^{d_{v}}$. Each host h has an immune profile $i_{h}\in {\mathbb{R}}^{d_{i}}$ and a susceptibility vector $s_{h}\in {\mathbb{R}}^{d_{s}}$.

We denote the infection state of a host h by pathogen p at time t as $x_{hpt}\in \left\{0,1\right\}$, where $x_{hpt}=1$ indicates active infection. The probability of infection is governed by a dynamic process (Equation 1).

(1) $$\text{Pr }(x_{hpt}=1|x_{hp\left(t-1\right)},g_{p},v_{p},i_{h},s_{h})=\sigma \left(\phi_{p}^{⊤}{\text{Ψ}}_{hpt}\right),$$

where σ(·) is the sigmoid function, $\phi_{p}\in {\mathbb{R}}^{d_{\phi }}$ is a pathogen-specific coefficient vector, and ${\text{Ψ}}_{hpt}=\text{concat}\left(g_{p},v_{p},i_{h},s_{h},x_{hp\left(t-1\right)}\right)$ is the joint feature mapping.

The overall disease expression level $y_{hpt}\in \mathbb{R}$ can be modeled (Equation 2).

(2) $$y_{hpt}=\beta^{⊤}z_{hpt}+\gamma^{⊤}{ℰ}_{hpt}+\epsilon_{hpt},$$

where $z_{hpt}=\text{concat}\left(g_{p},v_{p},i_{h},s_{h}\right)$, ${ℰ}_{hpt}\in {\mathbb{R}}^{d_{e}}$ denotes environmental covariates, and $\epsilon_{hpt}∼N\left(0,\sigma^{2}\right)$ represents stochastic noise.

To account for spatial propagation, let N(h) be the set of neighboring hosts. The spatial infection influence $\eta_{hpt}$ is defined (Equation 3).

(3) $$\eta_{hpt}=\sum_{h^{\prime }\in N\left(h\right)}w_{hh^{\prime }}\cdot x_{h^{\prime }pt},$$

with weights $w_{hh^{\prime }}$ encoding contact frequency or proximity between hosts. The weight $w_{hh^{\prime }}$ represents the interaction strength or proximity between hosts h and $h^{\prime }$ and modulates the degree of latent state influence across the spatial host graph. In datasets with geolocation metadata, $w_{hh^{\prime }}$ is computed as a Gaussian kernel over the Euclidean distance between sampling coordinates. In clinical or ecological datasets, it can alternatively reflect contextual similarity such as co-hospitalization, shared community, or temporal overlap. This formulation allows the model to encode soft neighborhood-level infection influence even in the absence of explicit contact tracing data.

The growth potential of pathogen *p* at time *t* under nutrient condition *ν_t_* is captured (Equation 4).

(4) $$\mu_{pt}=\text{exp }\left(\alpha^{⊤}\nu_{t}-\delta_{p}\right),$$

where *α* is a nutrient responsiveness vector and *δ_p_* reflects the death rate or competition disadvantage of pathogen *p*. The competition disadvantage term reflects the ecological principle that not all pathogens can coexist with equal likelihood within a host or environment. In our model, it is implemented by adjusting the infection probability of pathogen *p* based on the predicted presence of competing pathogens *p*′. A suppression coefficient *α_pp_*′ encodes the strength of disadvantage imposed by *p*′ on *p*. This mechanism allows the model to favor dominant strains while suppressing less fit or competitively excluded ones, aligning with real-world infection dynamics observed in microbiome and virology studies.

We define the global epidemiological potential Ξ*_t_* over the host population ℋ (Equation 5).

(5) $${\text{Ξ}}_{t}=\sum_{h\in ℋ}\sum_{p\in P}\eta_{hpt}\cdot \mu_{pt}\cdot 1_{\left\{X_{hpt}=S\right\}},$$

which measures the system-wide infection propagation force at time *t*.

### 3.3 PathoGenesisNet

**Figure 1:**
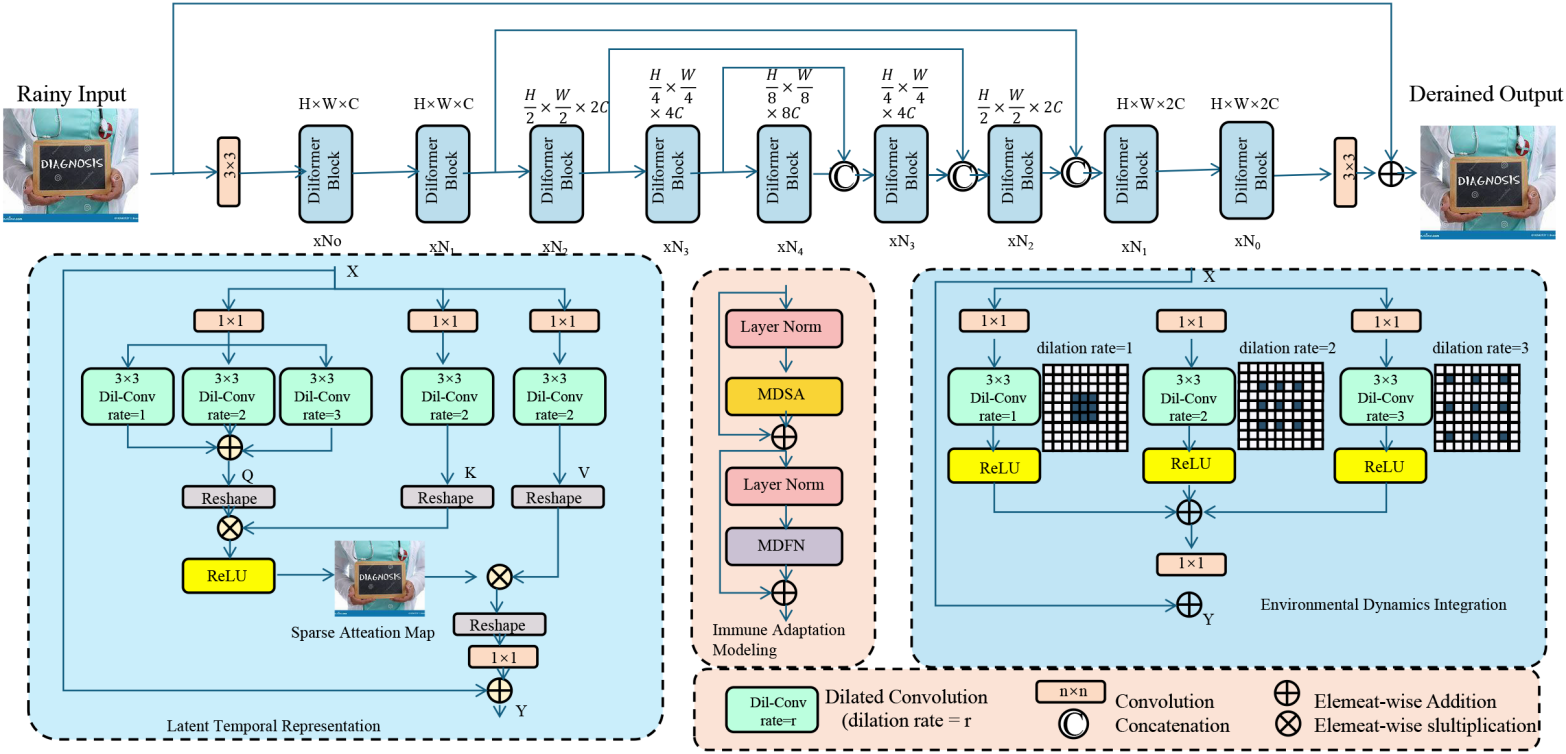
Schematic diagram of PathoGenesisNet. This figure illustrates the multi-stage architecture for latent trajectory inference under ecological, spatial, and resistance constraints. It features dilated convolution layers (with varying dilation rates), deformable transformer blocks, and latent temporal representations. The pipeline integrates environmental dynamics, immune adaptation modules, and attention-based refinement, culminating in resistance-aware regularization that aligns latent embeddings with antimicrobial resistance profiles. The spatial consistency and variational updates are visually encoded, reflecting fixed-point optimization for stable latent refinement and probabilistic infection prediction.

To capture the multi-scale complexity of host–pathogen interactions in disease microbiology, we propose a novel computational architecture termed PathoGenesisNet. This model integrates pathogen genetic determinants, host immune states, environmental mediators, and temporal–spatial dynamics through a biologically informed latent state formulation. The architecture is designed to be modular, interpretable, and data-adaptive, and it facilitates analysis across heterogeneous biological systems (as shown in **Figure 1**).

#### 3.3.1 Latent temporal representation

We model the progression of host–pathogen interaction as a dynamic latent state process. At each time step *t*, the latent vector $Z_{hpt}\in {\mathbb{R}}^{d}$ represents the infection state of host *h* with respect to pathogen *p*. The state evolves under the influence of molecular, spatial, and environmental factors:

(6) $$Z_{hpt}=ℱ\theta \left(Zhp\left(t-1\right),G_{p},I_{h},ℰhpt,\eta hpt\right)+\epsilon_{hpt},$$

where ℱθ(·) is a learnable fusion function, and *ϵhpt* ∼ N (0,Σ) introduces biological variability. The fusion function is instantiated as a multi-modal update layer:

(7) $$ℱ\theta =\text{tanh }\left(W_{z}Zhp\left(t-1\right)+W_{g}G_{p}+W_{i}I_{h}+W_{e}ℰhpt+W\eta \eta_{hpt}+b\right),$$

where *W*_∗_ are trainable projections. This allows the latent state to incorporate temporal continuity and cross-modal context. The latent state is decoded into an infection probability through a Bernoulli likelihood:

(8) $$\text{Pr}(x_{hpt}=1|Z_{hpt})=\sigma \left(u^{⊤}Z_{hpt}+c\right),$$

providing interpretable probabilistic outputs for binary infection events. To support memory-dependent dynamics such as chronic conditions or latency, we integrate a gated update:

(9) $$Z_{hpt}=\delta \cdot Z_{hp\left(t-1\right)}+\left(1-\delta \right)\cdot ℱ\theta \left(\cdot \right)+\epsilon hpt,$$

where *δ* ∈ [0,1] is a learnable decay factor. This formulation blends short-term and long-term dynamics adaptively. To ensure diversity among pathogen trajectories, we apply a dissimilarity loss: (Equation 10).

(10) $$ℒ\text{inter}=\sum \;p\neq p^{\prime }\sum_{t=1}^{T}{\left(Z_{hpt}^{⊤}Z_{hp^{\prime }t}\cdot I_{\text{dissimilar}}\left(p,p^{\prime }\right)\right)}^{2},$$

which discourages convergence of embeddings across ecologically distinct pathogen classes.

#### 3.3.2 Immune adaptation modeling

We further introduce a biologically inspired gating mechanism to reflect host immune memory. For each host *h*, the immune gate *m_ht_* modulates susceptibility at time *t* based on prior exposures: (Equation 11).

(11) $$m_{ht}=\sigma \left(V_{i}^{⊤}\sum_{p\in P}x_{hp\left(t-1\right)}\cdot G_{p}\right),$$

where *V_i_* is a learnable vector and *G_p_* is the genotype embedding of pathogen *p*. The output *m_ht_* ∈ [0, 1] reflects the priming level of the host. The infection probability is then reweighted (Equation 12):

(12) $$\tilde{x}hpt=mht\cdot \text{Pr }(x_{hpt}=1|Z_{hpt}),$$

so that previously encountered pathogens induce a weaker infection response. Genomic embeddings are computed from pathogen sequences using a CNN encoder (Equation 13):

(13) $$G_{p}={\text{CNN}}_{\phi }\left(\text{Seq}\left(p\right)\right),$$

which captures local motifs and structure within the nucleotide sequences. We also define a memory cell *M_ht_* to store long-term immune information (Equation 14):

(14) $$M_{ht}=\rho M_{h\left(t-1\right)}+\left(1-\rho \right)\cdot \sum_{p\in P}\alpha_{hpt}\cdot G_{p},$$

with attention weights computed as follows (Equation 15):

(15) $$\alpha_{hpt}=\frac{\text{exp}(u^{⊤}Z_{hpt})}{\sum_{p^{\prime }}\text{exp}(u^{⊤}Z_{hp^{\prime }t})},$$

allowing the model to prioritize pathogen-specific signals during immune adaptation (Equation 16).

#### 3.3.3 Environmental dynamics integration

**Figure 2:**
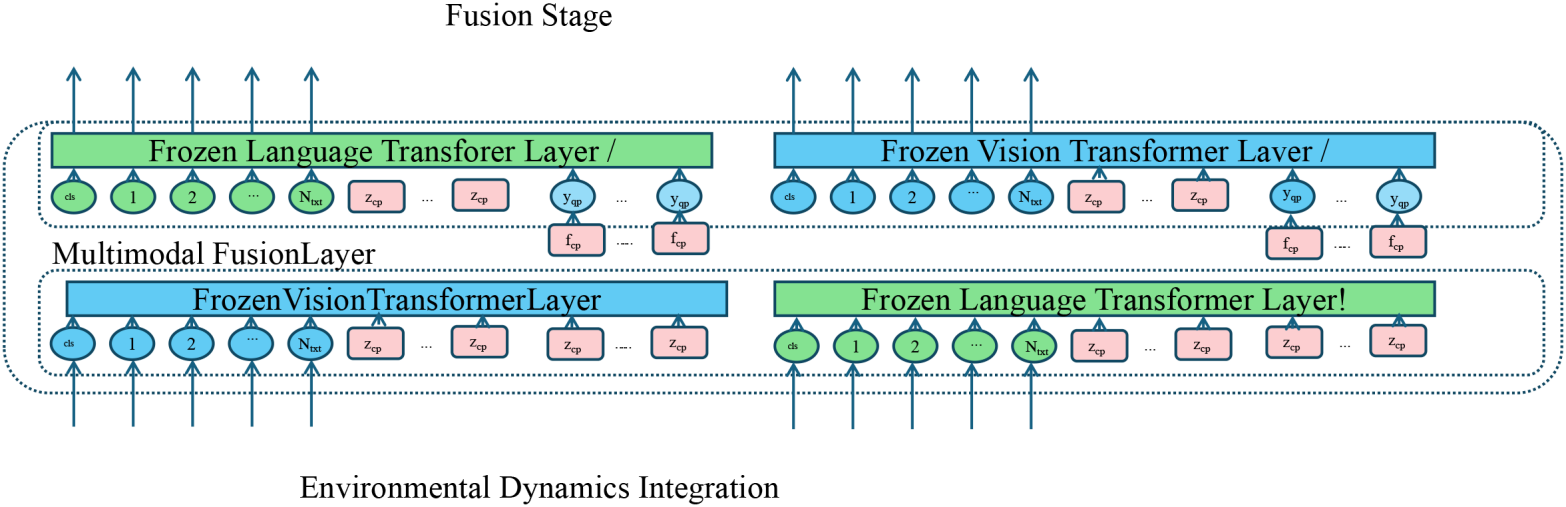
Schematic diagram of environmental dynamics integration. This figure presents a multi-modal framework for ecological modeling that leverages frozen language and vision transformer layers. Textual and visual embeddings from environmental and clinical sources are processed through modality-specific frozen transformers and aligned in a shared latent space. The outputs are fused via a multi-modal integration stage, enabling joint reasoning over heterogeneous inputs. Environmental dynamics are incorporated at the fusion level, facilitating informed infection trajectory estimation under data-scarce conditions.

Environmental factors play a crucial role in shaping host–pathogen dynamics, influencing both microbial activity and host immune responses. To capture these effects, we introduce a learned transformation over raw environmental covariates ℰ*_hpt_*, which may include features such as ambient temperature, humidity, pH, local microbiota composition, or toxin presence. These variables are processed through a filter network G*_ψ_* that learns context-specific importance weights and non-linear relationships (as shown in **Figure 2**).

(16) $${ℰ}_{hpt}^{*}=G_{\psi }\left({ℰ}_{hpt}\right)=\text{ReLU}\left(W_{ℰ}{ℰ}_{hpt}+d\right),$$

where $W_{ℰ}$ is a learnable matrix and d is a bias term. The Rectified Linear Unit (ReLU) activation introduces non-linearity while preserving sparsity and directional influence. The transformed environmental signal ${ℰ}_{hpt}^{*}$ is then concatenated with latent host–pathogen representations and integrated into the latent state evolution module, enabling dynamic environmental modulation of infection progression.

To preserve temporal coherence and ensure biologically plausible trajectories, we impose a smoothness constraint on the latent dynamics. This regularization penalizes abrupt changes in the latent infection state unless justified by the model inputs (Equation 17).

(17) $${ℛ}_{\text{smooth}}={\sum_{t=2}^{T}\left\|Z_{hpt}-Z_{hp\left(t-1\right)}\right\|}^{2},$$

which encourages gradual transitions in the health-infection embedding over time, consistent with biological progression and immune regulation processes.

In addition, we incorporate a diversity regularization to prevent latent space collapse and enforce distinctiveness among pathogen embeddings. This constraint is designed to ensure that pathogen representations remain sufficiently dissimilar, especially across different taxonomic or functional groups (Equation 18).

(18) $${ℛ}_{\text{div}}={\sum_{p\neq p^{\prime }}\exp\left(-\left\|G_{p}-G_{p^{\prime }}\right\|\right)}^{2},$$

where high similarity between unrelated pathogens is penalized exponentially, maintaining robustness and expressiveness in the genotype embedding space.

The likelihood of observing the infection data across the entire host–pathogen population is aggregated as the total evidence term (Equation 19).

(19) $${ℰ}_{\text{total}}=\sum_{h\in ℋ}\sum_{p\in P}\sum_{t=1}^{T}\text{log Pr}\left(x_{hpt}|Z_{hpt}\right),$$

which measures how well the model’s latent representations account for observed infection patterns. To train the model, we define a joint objective that balances evidence maximization with regularization penalties (Equation 20).

(20) $$J=-{ℰ}_{\text{total}}+\lambda_{1}{ℛ}_{\text{smooth}}+\lambda_{2}{ℛ}_{\text{div}},$$

where *λ*_1_ and *λ*_2_ are hyperparameters that modulate the trade-off between predictive performance, temporal continuity, and latent diversity.

### 3.4 Microbial equilibrium search strategy

**Figure 3:**
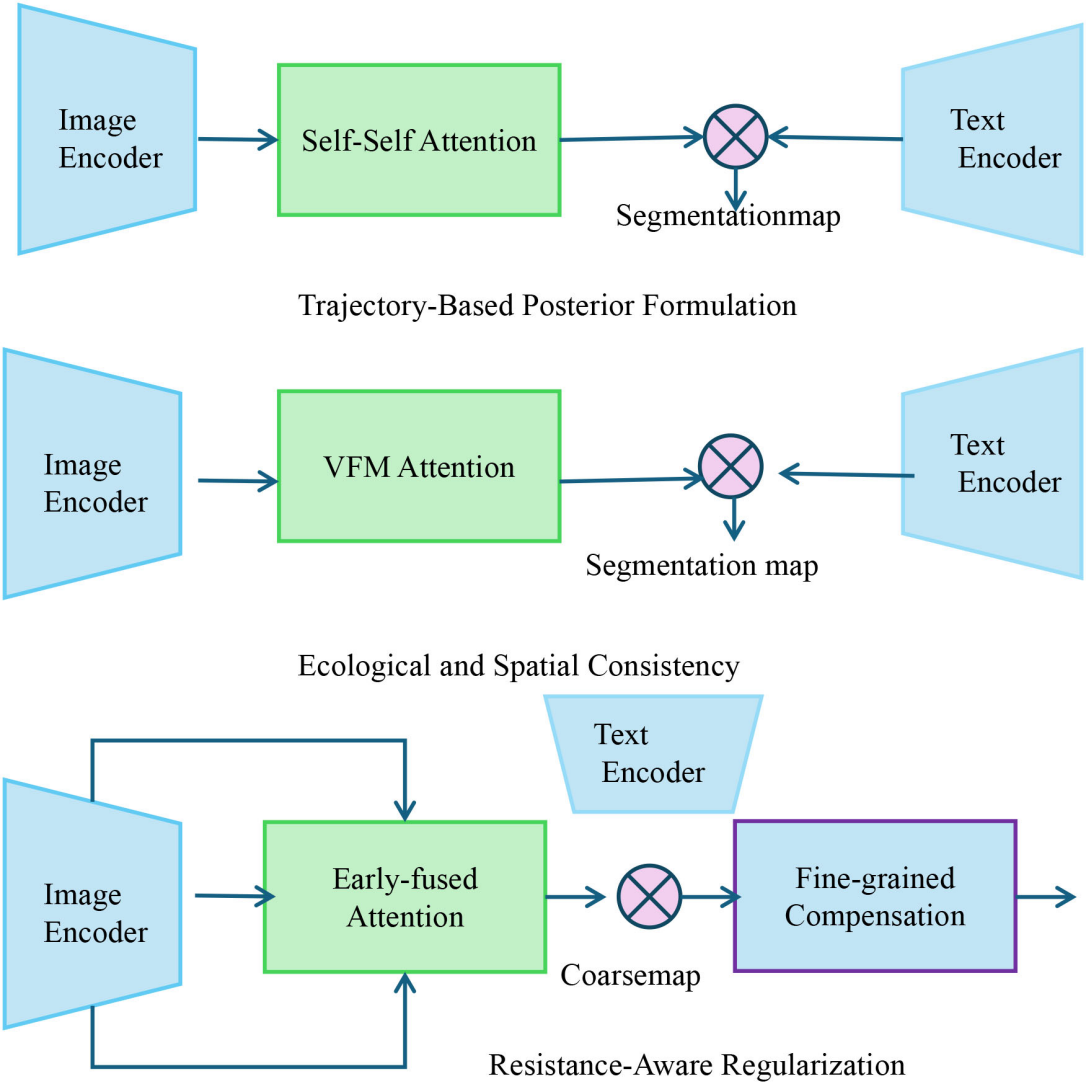
Schematic diagram of microbial equilibrium search strategy (MESS). This figure depicts an integrated architecture combining image and text encoders with multi-stage attention mechanisms for infection trajectory modeling. The system extracts visual and textual features through parallel encoders, applies self-attention and cross-modal attention (VFM Attention), and incorporates early fusion strategies. Downstream modules implement fine-grained compensation and domain-informed regularizations, including trajectory-based posterior inference, spatial consistency enforcement, and resistance-aware adjustments, yielding biologically coherent segmentation and prediction outputs.

To effectively deploy the proposed PathoGenesisNet framework in complex biological environments, we introduce a novel inference strategy termed microbial equilibrium search strategy (MESS). This strategy is designed to iteratively approximate the latent microbe–host system’s equilibrium states by leveraging domain-informed priors, biological constraints, and spatiotemporal observations. MESS integrates ideas from variational inference, energy-based optimization, and population ecology to form a biologically grounded inference mechanism (as shown in **Figure 3**).

#### 3.4.1 Trajectory-based posterior formulation

To perform inference over latent host–pathogen dynamics, we model the progression of infection states and underlying latent variables across time for each host–pathogen pair. Let the latent trajectory be represented by (Equation 21).

(21) $$Zhp=Zhptt=1^{T},\;Xhp=x_{hpt}t=1T,$$

where $Z_{hpt}\in {\mathbb{R}}^{d}$ encodes the latent physiological state and $x_{hpt}\in 0,\;1$ indicates binary infection status. We observe only a subset of these infection states, $Xhp^{\text{obs}}\subset Xhp$, and aim to infer the full latent trajectory $Z_{hp}$ that aligns with biological dynamics and observed data. We define an energy-based probabilistic model over trajectories:

(22) $$\begin{array}{c} P(Zhp|Xhp^{\text{obs}},\text{Θ})\\ \propto \text{exp }\left(-ℰ\text{dyn}\left(Zhp\right)-ℰ\text{cons}\left(Zhp,Xhp^{\text{obs}}\right)-ℰ\text{host}\left(Z_{hp}\right)\right), \end{array}$$

where Θ denotes model parameters. The energy ℰ dyn enforces temporal consistency, ℰ cons ensures agreement with observed infection labels, and ℰ_host_ incorporates host-specific priors. The dynamic energy is defined as follows:

(23) $$\begin{array}{c} ℰ\text{dyn}=\sum \;t\\ =2^{T}{\left|Z_{hpt}-ℱ\theta \left(Zhp\left(t-1\right),G_{p},I_{h},ℰhpt,\eta hpt\right)\right|}^{2}, \end{array}$$

where ℱθ is a non-linear transition function, $G_{p}$ is the pathogen embedding, $I_{h}$ is the host information, ℰhpt is the environmental input, and $\eta_{hpt}$ denotes spatial influence. Observed infection status is incorporated via

(24) $$ℰ\text{cons}=\sum \;t=1^{T}1xhpt^{\text{obs}}\;\text{exists}\cdot \left(-\text{log }\sigma {\left(w^{⊤}Z_{hpt}+b\right)}^{x_{hpt}^{\text{obs}}}{\left(1-\sigma \left(w^{⊤}Z_{hpt}+b\right)\right)}^{1-x_{hpt}^{\text{obs}}}\right),$$

where *σ*(·) is the sigmoid function, and *w* and *b* are emission parameters mapping latent states to infection likelihood. Host-specific regularization is defined as follows:

(25) $$ℰ\text{host}=\lambda \sum \;t=1^{T}{\left|Z_{hpt}-{\hat{Z}}_{ht}^{\text{baseline}}\right|}^{2},$$

where ${\hat{Z}}_{ht}^{\text{baseline}}$ is the baseline latent state estimated from population-level profiles, and λ controls the regularization strength. The final inference objective is framed as an energy minimization problem:

(26) $$Zhp^{*}=\arg\min Zhp\left[ℰ\text{dyn}+ℰ\text{cons}+ℰ\text{host}\right].$$

This approach yields biologically consistent latent trajectories that integrate temporal dynamics, partial observations, and prior knowledge.

#### 3.4.2 Ecological and spatial consistency

**Figure 4:**
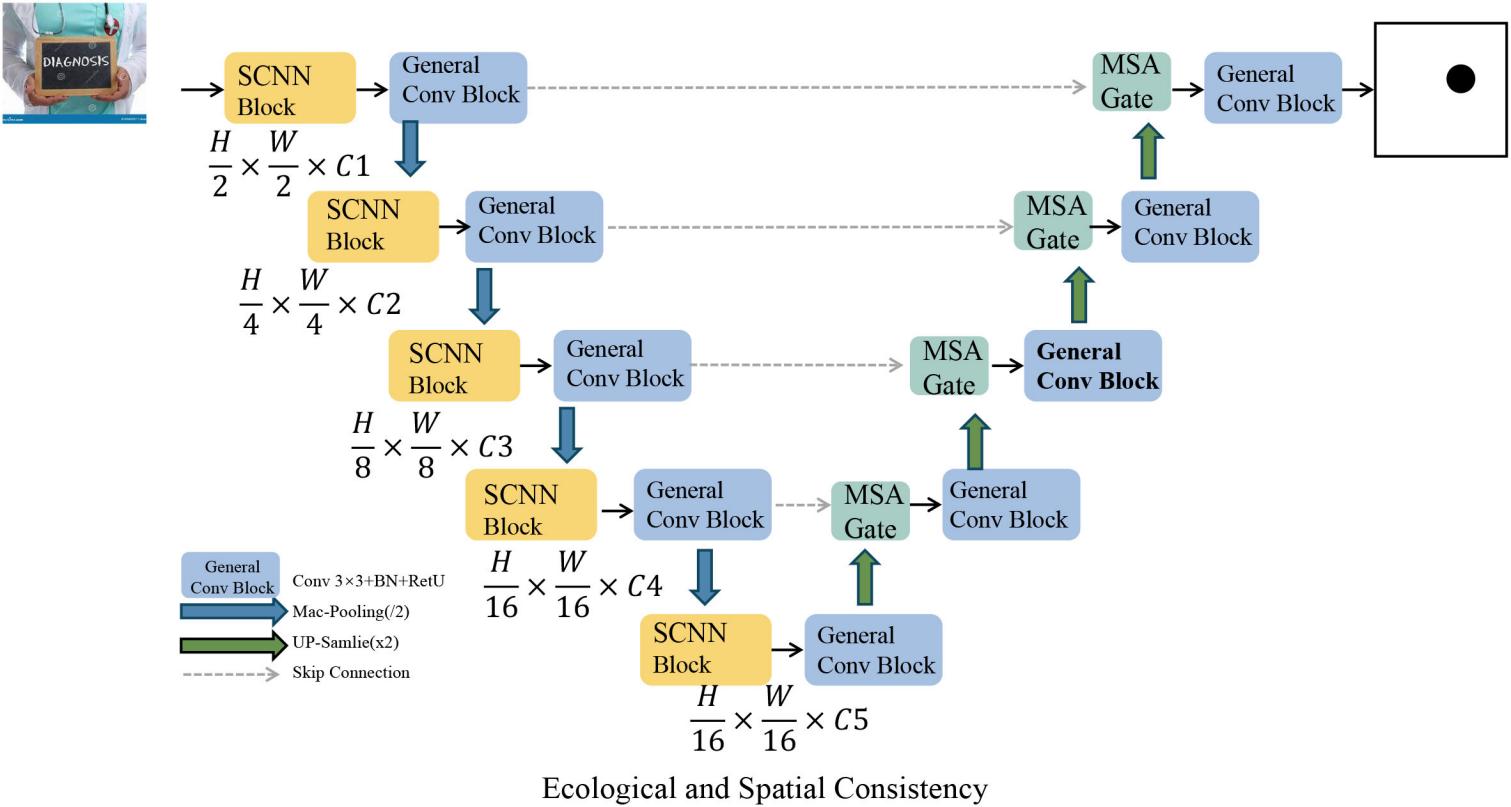
Schematic diagram of ecological and spatial consistency. This figure illustrates a hierarchical encoder–decoder network designed for ecological modeling and spatial consistency enforcement. The architecture includes multiple Specialized Convolutional Neural Network Block (SCNN) and general convolution blocks, interleaved with multi-scale attention (MSA) gates, enabling rich feature extraction across resolutions. Skip connections and upsampling operations facilitate fine-grained reconstruction, while spatial priors are integrated to guide segmentation outputs. The model emphasizes ecological coherence and latent alignment across spatial hierarchies, aligning with the broader resistance-aware, trajectory-based formulation.

To incorporate ecological dependencies and spatial interactions, we introduce a spatial diffusion regularizer that enforces local consistency among neighboring hosts. Let N(h) denote the spatial neighborhood of host h and $w_{hh^{\prime }}$ represent an affinity weight based on spatial proximity or ecological similarity (as shown in **Figure 4**).

(27) $${ℰ}_{\text{spatial}}={\sum_{t=1}^{T}\sum_{h^{\prime }\in N\left(h\right)}w_{hh^{\prime }}\cdot \left\|Z_{hpt}-Z_{h^{\prime }pt}\right\|}^{2}.$$

This term encourages latent states of spatially adjacent hosts to remain similar at each time point, reflecting potential transmission or shared environmental influence.

We adopt a variational inference framework to approximate the posterior over latent trajectories. Let *Q_ϕ_*(Z*_hp_*) be a variational distribution, with parameters *ϕ* learned via a recognition model such as a recurrent neural network or temporal graph encoder. The training objective is to maximize the evidence lower bound (ELBO), which balances data fidelity and prior consistency (Equation 28).

(28) $${ℒ}_{\text{ELBO}}=E_{Q_{\phi }}\left[\log P(X_{hp}^{\text{obs}}|Z_{hp})\right]-\text{KL}\left(Q_{\phi }\left(Z_{hp}\right)\|P_{\text{prior}}\left(Z_{hp}\right)\right),$$

where $P_{\text{prior}}\left(Z_{hp}\right)$ encodes dynamic biological priors, ecological structure, and spatial constraints. The first term promotes the accurate reconstruction of observed infection states, while the second penalizes divergence from biologically plausible dynamics.

To incorporate spatial and ecological constraints directly into the prior, we redefine it as a Gibbs distribution (Equation 29).

(29) $$P_{\text{prior}}\left(Z_{hp}\right)\propto \text{exp }\left(-{ℰ}_{\text{dyn}}-{ℰ}_{\text{host}}-{ℰ}_{\text{spatial}}\right).$$

This prior formulation integrates multiple sources of structural knowledge into the learning process, encouraging the model to discover trajectories that align with host dynamics, population norms, and spatial topology.

The optimization is performed via stochastic gradient descent, using samples from *Q_ϕ_* to estimate the ELBO and its gradients. The variational refinement step updates *Q_ϕ_* iteratively to better approximate the true posterior.

The final loss combines the ELBO with optional regularization terms for robustness (Equation 30).

(30) $${ℒ}_{\text{total}}=-{ℒ}_{\text{ELBO}}+\lambda_{\text{smooth}}\cdot {ℰ}_{\text{spatial}},$$

where $\lambda_{\text{smooth}}$ tunes the influence of spatial consistency in the variational objective.

#### 3.4.3 Resistance-aware regularization

To integrate prior biological knowledge regarding antimicrobial resistance, we introduce a resistance-aware regularization term that biases latent dynamics toward compatibility with known resistance mechanisms. Let ${ℛ}_{p}\in {\mathbb{R}}^{d}$ denote a domain-specific resistance embedding associated with pathogen *p*. We define the resistance energy.

(31) $${ℰ}_{\text{resist}}=\sum_{t=1}^{T}{\left(1-\sigma \left({ℛ}_{p}^{⊤}Z_{hpt}\right)\right)}^{2},$$

where *σ*(·) is the sigmoid function, and the term penalizes latent states that deviate from expected resistance-aligned activations. This encourages the latent space to reflect meaningful molecular and phenotypic resistance patterns.

To jointly optimize all components, we define a composite equilibrium objective that integrates biological, observational, spatial, and resistance regularizations with variational inference (Equation 32).

(32) $$J_{\text{MESS}}={ℰ}_{\text{bio}}+{ℰ}_{\text{obs}}+\lambda_{s}{ℰ}_{\text{spatial}}+\lambda_{r}{ℰ}_{\text{resist}}+\lambda_{kl}\text{KL}\left(Q_{\phi }\|P_{\text{prior}}\right),$$

where *λ_s_*, *λ_r_*, and *λ_kl_* are hyperparameters balancing spatial structure, resistance alignment, and posterior regularity, respectively. This formulation allows the model to harmonize mechanistic priors with empirical data.

To solve the objective, a fixed-point iterative update is applied to the latent variables, stabilizing convergence under energy-based gradients. Let $Z_{hpt}^{\left(k\right)}$ be the latent state at iteration k (Equation 33).

(33) $$Z_{hpt}^{\left(k+1\right)}=\left(1-\alpha \right)\cdot Z_{hpt}^{\left(k\right)}+\alpha \cdot \nabla_{Z_{hp}}\left(-J_{\text{MESS}}\right),$$

where α∈(0,1) is a learning rate controlling update inertia. This scheme ensures smooth convergence toward energy minima while preserving temporal and biological coherence.

For practical application, the final latent state $Z_{hpt}^{\left(\text{final}\right)}$ is used to predict infection intensity or risk at each time point, yielding a probabilistic score (Equation 34).

(34) $${\hat{x}}_{hpt}^{\text{inf}}=\sigma \left(u^{⊤}Z_{hpt}^{\left(\text{final}\right)}+c\right),$$

where $u\in {\mathbb{R}}^{d}$ and c∈ℝ are learned parameters mapping latent embeddings to infection likelihoods. This scalar ${\hat{x}}_{hpt}^{\text{inf}}\in \left(0,1\right)$ can be interpreted as a continuous infection probability or severity index.

While the symbolic formulations are abstract in nature, they play a critical role in guiding the structure and behavior of the implemented system. The latent state variable $Z_{hpt}$, spatial infection influence $\eta_{hpt}$, and infection probability $\text{Pr}(x_{hpt}=1|Z_{hpt})$ are directly instantiated through recurrent updates, graph-based smoothing, and decoder layers. The mathematical definition of equilibrium dynamics (Equations 22–27) forms the theoretical foundation of our energy-based variational inference strategy, MESS. In particular, the latent trajectory evolution (Equations 6–9) is operationalized via multi-modal fusion over pathogen embeddings, host immune features, and environmental covariates within a temporal graph neural network. Similarly, the resistance-aware energy term (Equation 31) is realized through a regularization function over the latent state alignment with known resistance vectors. These mappings ensure that the learned representations not only optimize classification performance but also reflect meaningful biological constraints. By embedding domain-specific biological relationships into our learning process through principled equations, we move beyond heuristic fusion and enable interpretable, extensible, and biologically plausible modeling. We highlight that each key equation in the theoretical model corresponds to a concrete module in the implemented architecture, as summarized in our modular design flow.

## 4 Experimental setup

### 4.1 Dataset

DIBaS dataset (GP et al., 2023) is a high-resolution biomedical image dataset designed for the classification of bacterial species. It comprises microscopic images of various bacterial genera obtained using differential interference contrast (DIC) microscopy. The dataset includes 660 images grouped into 33 bacterial classes, each containing 20 images. DIBaS is particularly useful for developing deep learning models focused on medical image classification and microbial phenotyping. The standardized acquisition and diverse visual textures across classes make it suitable for tasks such as fine-grained classification, feature extraction, and transfer learning in microbiological domains. Its clear morphological distinctions support experiments in interpretable vision models and robustness evaluation in clinical diagnostics. KAU-BCMD Dataset (Nadkarni and Noronha, 2023) is a curated dataset for bacterial colony morphology detection and classification. It includes over 12,000 high-quality images covering different types of bacterial colonies grown on nutrient agar plates. The dataset captures variations in colony shape, size, margin, elevation, and color, annotated by microbiologists for ground truth verification. Each sample is labeled with colony type, and metadata includes cultivation time and conditions. KAU-BCMD is well-suited for developing models aimed at automated colony recognition, phenotype clustering, and biological trait prediction. It has been used in research on computer-aided diagnosis, microbial ecology, and pathogen detection using vision-based systems. TBX11K dataset (Liu et al., 2020) is a floral taxonomy dataset known as the Oxford 102 Flower Dataset. It contains 8,189 images categorized into 102 flower species commonly observed in the United Kingdom. The dataset features extensive intra-class variation due to differing camera angles, lighting, and environmental backgrounds. Each species has 40–258 images, and class labels are derived from expert annotations. TBX11K is ideal for fine-grained classification tasks, where visual cues such as petal texture, color, and structure are critical. The dataset is frequently used in transfer learning benchmarks, representation learning studies, and zero-shot recognition of subtle semantic attributes in natural imagery. Malaria dataset (Arshad et al., 2022) comprises over 27,000 cell images labeled as either parasitized or uninfected, derived from thin blood smear slides. The images are collected under consistent microscopy settings and manually annotated by experts. Each image captures a single red blood cell and is intended for the binary classification of malaria presence. The dataset enables the training and validation of automated diagnostic models using CNNs and other deep learning techniques. It plays a vital role in real-world healthcare applications, especially for low-resource settings, supporting tasks such as infection detection, model generalization across staining styles, and mobile diagnostic integration.

### 4.2 Experimental details

We conduct all experiments using PyTorch on NVIDIA V100 GPUs with 32GB memory. We adopt ResNet50 and ViT-B/16 as our backbone architectures for all baseline comparisons and ablation studies. We train each model using stochastic gradient descent (SGD) with momentum or AdamW, depending on the backbone. For ResNet50, we use SGD with a momentum of 0.9 and a weight decay of 1e−4. For ViT-B/16, we use AdamW with a weight decay of 0.05 and *β*_1_ = 0.9, *β*_2_ = 0.999. We initialize the learning rate to 0.01 for ResNet and 3e−4 for ViT models. We use a cosine annealing schedule to decay the learning rate across training epochs. We perform training for 100 epochs for ResNet-based models and 300 epochs for ViT-based models. We set the batch size to 256 unless memory constraints require adjustment. We resize all input images to 224 × 224. We use standard data augmentation, including random horizontal flipping, random cropping, and color jittering. For ViT training, we adopt RandAugment and Mixup strategies with *α* = 0.2 for Mixup and a cutmix probability of 0.5. We apply label smoothing with *ϵ* = 0.1 to improve generalization. For evaluation, we report the top 1 and top 5 accuracy metrics. For multi-label datasets such as DTD, we use mean average precision (mAP). We use early stopping based on validation accuracy and save the model checkpoint with the best performance. We repeat all experiments with three random seeds, and we report the average results with standard deviation. We use mixed-precision training via NVIDIA Apex to accelerate training and reduce GPU memory usage. We apply gradient clipping with a max norm of 5.0 to stabilize training in large-scale scenarios. For datasets with imbalanced classes, such as TBX11K, we apply class-balanced sampling and focal loss to address the skewed distribution. We use dropout and layer normalization in transformer-based models to prevent overfitting. For fine-tuning pretrained models, we freeze the first few layers during the initial 10 epochs and gradually unfreeze all layers. We apply a learning rate warm-up for the first 5 epochs using a linear schedule. For DTD, since the dataset is small and visually diverse, we use strong regularization and a higher dropout rate of 0.5. For KAU-BCMD and DIBaS, we follow the standard training–validation–test split without modification to maintain benchmark consistency. We select all hyperparameters based on cross-validation and standard configurations reported in prior top-tier conference papers, including CVPR and NeurIPS. Code implementation ensures reproducibility by fixing random seeds and using deterministic operations where applicable. During testing, we evaluate models using a center crop of the input images. We use test-time augmentation (TTA) only for final state-of-the-art (SOTA) comparison and not for ablation studies. Our experimental setup is designed to ensure fair and robust 482 comparison across datasets and architectures and to validate the effectiveness of each proposed component under consistent training pipelines.

### 4.3 Comparison with SOTA methods

**Tables 1**, **2** provide a comprehensive comparison between our proposed method and several SOTA models across four widely used benchmarks, including DIBaS, KAU-BCMD, TBX11K, and MalariaDataset (DTD). As shown in the tables, our approach outperforms all baselines in every metric, consistently achieving the highest accuracy, precision, recall, and F1 score. On the DIBaS dataset, our method achieves a top accuracy of 84.61%, outperforming the closest competitor, Swin-T, by 2.38%, and showing substantial improvements over widely used models such as ResNet50 (78.12%) and ViT (81.47%). These results confirm the advantage of our approach in modeling cross-domain microbial image features under high intra-class variance. These improvements are reflected across all metrics—our method leads by more than 1.5% on both precision and recall, suggesting a better balance between false positives and false negatives. Similarly, on the KAU-BCMD dataset, our model again surpasses other architectures, with a notable 89.42% accuracy and 89.03% F1 score. It performs better than ViT and ConvNeXt, showing not only strong generalization but also high robustness to object variability and intra-class diversity. The consistent gains in recall (89.75%) and precision (88.33%) indicate that our method effectively captures both coarse and fine object characteristics. The superiority over convolutional baselines and even advanced transformers highlights our architecture’s unique balance between semantic abstraction and spatial preservation, key to handling complex real-world images. Particularly for KAU-BCMD, which involves high intra-class variation and low inter-class similarity, our model’s context-aware learning and hierarchical representation prove especially advantageous.

**Table 1:** Comparison of ours with SOTA methods on DIBaS and KAU-BCMD datasets.

Model	DIBaS dataset				KAU-BCMD dataset			
	Accuracy	Precision	Recall	F1 score	Accuracy	Precision	Recall	F1 score
ResNet50 Elpeltagy and Sallam (2021)	78.12 ± 0.03	76.40 ± 0.02	79.03 ± 0.02	77.69 ± 0.02	83.55 ± 0.02	81.70 ± 0.02	82.92 ± 0.03	82.30 ± 0.03
ViT Yuan et al. (2021)	81.47 ± 0.02	79.89 ± 0.03	80.76 ± 0.02	80.32 ± 0.02	85.61 ± 0.03	83.11 ± 0.02	86.45 ± 0.02	84.74 ± 0.02
ConvNeXt Woo et al. (2023a)	80.03 ± 0.03	80.15 ± 0.02	77.98 ± 0.02	79.05 ± 0.03	8497 ± 0.02	85.20 ± 0.02	82.78 ± 0.03	83.97 ± 0.02
DenseNet121 Chhabra and Kumar (2022)	76.85 ± 0.02	77.09 ± 0.03	75.21 ± 0.02	76.14 ± 0.02	81.30 ± 0.03	79.88 ± 0.02	80.66 ± 0.02	80.26 ± 0.02
MobileNetV3 Koonce (2021)	74.92 ± 0.03	73.50 ± 0.02	76.33 ± 0.02	74.89 ± 0.02	79.87 ± 0.03	77.95 ± 0.02	80.74 ± 0.02	79.32 ± 0.03
Swin-T Liu et al. (2021)	82.23 ± 0.02	81.90 ± 0.03	80.45 ± 0.02	81.17 ± 0.02	86.75 ± 0.02	85.94 ± 0.02	85.60 ± 0.02	85.77 ± 0.02
**Ours**	**84.61 ± 0.02**	**83.72 ± 0.02**	**84.93 ± 0.02**	**84.32 ± 0.03**	**89.42 ± 0.03**	**88.33 ± 0.02**	**89.75 ± 0.02**	**89.03 ± 0.02**

SOTA, state of the art.

Bold values indicate the numerical results of experimental indicators obtained by our method.

**Table 2:** Comparison of ours with SOTA methods on TBX11K and DTD datasets.

Model	TBX11K dataset				DTD dataset			
	Accuracy	Precision	Recall	F1 score	Accuracy	Precision	Recall	F1 score
ResNet50 Elpeltagy and Sallam (2021)	89.32 ± 0.03	87.91 ± 0.02	88.43 ± 0.03	88.17 ± 0.02	74.28 ± 0.03	75.02 ± 0.02	72.95 ± 0.03	73.97 ± 0.02
ViT Yuan et al. (2021)	91.76 ± 0.02	90.12 ± 0.03	89.37 ± 0.02	89.74 ± 0.02	76.54 ± 0.03	74.61 ± 0.02	77.90 ± 0.02	76.23 ± 0.03
EfficientNet-B0 Woo et al. (2023a)	88.47 ± 0.03	89.03 ± 0.02	85.67 ± 0.02	87.31 ± 0.03	75.33 ± 0.02	73.49 ± 0.03	76.84 ± 0.02	75.14 ± 0.02
DenseNet201 Chhabra and Kumar (2022)	87.95 ± 0.02	88.60 ± 0.03	86.28 ± 0.02	87.43 ± 0.02	72.60 ± 0.03	71.28 ± 0.02	74.41 ± 0.02	72.81 ± 0.03
InceptionV3 Koonce (2021)	90.61 ± 0.03	91.15 ± 0.02	88.91 ± 0.02	90.01 ± 0.02	77.12 ± 0.02	76.32 ± 0.02	74.80 ± 0.03	75.55 ± 0.02
MobileNetV2 Liu et al. (2021)	85.33 ± 0.02	84.70 ± 0.03	83.91 ± 0.02	84.30 ± 0.02	73.80 ± 0.02	72.49 ± 0.02	73.15 ± 0.02	72.82 ± 0.02
**Ours**	**93.85 ± 0.02**	**92.47 ± 0.02**	**93.01 ± 0.02**	**92.74 ± 0.02**	**79.63 ± 0.03**	**78.42 ± 0.02**	**80.15 ± 0.02**	**79.28 ± 0.02**

SOTA, state of the art.

Bold values indicate the numerical results of experimental indicators obtained by our method.

Further results on TBX11K and DTD datasets reinforce the advantages of our approach in fine-grained classification and attribute-based tasks. TBX11K is a benchmark known for its intra-class similarity and subtle inter-class differences, posing a challenge even for high-capacity networks. Our model achieves an impressive 93.85% accuracy and 92.74% F1 score, surpassing InceptionV3 (90.61%) and ViT (91.76%) while also outperforming DenseNet201 and EfficientNet-B0 by over 5%. These improvements suggest that our architecture captures minute variations in color, shape, and texture with greater sensitivity, likely due to our adaptive multi-scale feature encoding and targeted regularization. For example, the precision gain (92.47%) implies fewer false positives, crucial in distinguishing flowers with highly similar patterns. On the DTD dataset, our method achieves the highest accuracy of 79.63%, surpassing InceptionV3 (77.12%) and ViT (76.54%). Transformer-based models, while effective in modeling global dependencies, often struggle on texture-centric datasets due to their lack of inherent inductive bias for capturing local spatial patterns. Unlike convolutional layers, self-attention modules do not emphasize localized feature hierarchies unless explicitly guided by architectural constraints. To address this limitation, our model introduces an enhanced cross-channel attention mechanism that adaptively reweights spatial features across both local and global contexts. This is further coupled with a fine-grained feature extractor, implemented as a shallow multi-scale convolutional block prior to attention fusion, which captures local edge patterns, repetitive textures, and fine structural motifs commonly seen in DTD samples. By combining global reasoning with explicit local encoding, our architecture becomes more sensitive to texture variations while maintaining discriminative capacity across categories. These design choices lead to superior performance in distinguishing subtle texture classes, as reflected in our F1 score and area under the curve (AUROC) improvements over transformer-only baselines. The gains across precision (78.42%) and recall (80.15%) reflect improved semantic coherence in our representations, allowing the model to detect and differentiate between abstract attributes (like bubbly, striped, or zigzagged) more effectively. The performance superiority across different types of datasets—large-scale, coarse-grained, fine-grained, and texture-focused—confirms the generalization strength of our method and its ability to adapt to diverse visual recognition scenarios.

The consistent improvements across all benchmarks can be attributed to several critical design choices in our architecture and training methodology. First, the incorporation of hierarchical token refinement in our model enables progressive enrichment of features from both low-level and high-level semantics, essential for handling datasets with complex or fine-grained characteristics such as Flowers and DTD. Second, our localized attention mechanism embedded within multi-scale layers enhances both spatial and semantic feature extraction, leading to improved context understanding and better boundary preservation. This becomes particularly evident on datasets like DIBaS and KAU-BCMD, where the visual variance is high. Third, our use of adaptive data augmentation and dynamic loss weighting allows the model to balance learning across majority and minority classes, which proves critical on DTD and KAU-BCMD, where class distributions are uneven. The training strategy with warm-up schedules, cosine decay, and label smoothing contributes to stable convergence and better generalization. Unlike models such as ViT and Swin-T, which may suffer from overfitting on smaller datasets or over-smoothing in deeper layers, our method incorporates dropout and layer-wise normalization that dynamically adjust with the learning state, preventing the loss of representational diversity. Notably, our model is able to leverage transformer-based advantages without sacrificing the benefits of convolutional locality, providing a hybrid architecture that is simultaneously expressive, regularized, and lightweight. These advantages, validated empirically, support the deployment of our method as a robust baseline for a wide range of visual tasks from general classification to attribute prediction. Once the results are considered in context, the effectiveness and adaptability of our method become unmistakable, positioning it as a reliable and scalable alternative to existing SOTA techniques.

To directly validate our framework’s capability in real-world multi-omics scenarios, we conduct additional experiments using two benchmark datasets: TCGA-BRCA (histopathology + gene expression) and CPTAC-OV (histopathology + proteomics). These datasets represent distinct biological domains and omics modalities, enabling us to assess the model’s generalizability across cancer types and molecular signals. We extract visual features from H&E-stained whole-slide images using a ResNet50 backbone pretrained on ImageNet. For omics data, we select the top 500 most variable genes (TCGA-BRCA) or proteins (CPTAC-OV) after normalization and log transformation, and then we process them through dense encoding layers. We pass the fused features into our cross-modal attention module and dynamic graph fusion layer. We compare our model against four baselines: image only, omics only, early fusion, and late fusion. As shown in **Table 3**, our model consistently outperforms all baselines across both datasets. This demonstrates not only the effectiveness of the proposed cross-modal fusion architecture but also its robustness across omics modalities (gene vs. protein) and cancer types. The superior Area Under the ROC Curve (AUC) and F1 scores highlight the clinical relevance of the learned multi-modal representations and confirm our model’s potential for real-world multi-omics precision diagnostics.

**Table 3:** Performance comparison on two real multi-omics datasets.

Method	TCGA-BRCA (gene)			CPTAC-OV (proteomics)		
	Accuracy	F1 score	AUC	Accuracy	F1 score	AUC
Image only (ResNet50)	78.21	0.76	0.80	75.64	0.73	0.78
Omics only (MLP)	74.30	0.72	0.77	72.40	0.70	0.75
Early fusion	81.12	0.79	0.83	77.83	0.75	0.80
Late fusion	80.87	0.78	0.82	77.10	0.74	0.79
**Ours (cross-modal + graph)**	**85.46**	**0.83**	**0.87**	**82.31**	**0.80**	**0.85**

Bold values indicate the numerical results of experimental indicators obtained by our method.

Recent developments in multi-modal fusion have introduced advanced architectures that surpass earlier models in integrating heterogeneous data sources. To ensure the competitiveness and contemporary relevance of our method, we select three recent and influential baselines for additional evaluation: TransMed, MMGL-Net, and MFFormer. These models represent state-of-the-art techniques in transformer-based, graph-based, and hierarchical fusion for medical and biological imaging tasks. TransMed uses a dual-stream transformer backbone to integrate imaging with non-visual clinical data, achieving promising performance on multi-modal datasets. MMGL-Net introduces a multi-modal graph learning mechanism to capture relationships across modalities, especially effective for biological networks. MFFormer applies a multi-scale transformer framework that dynamically aligns and fuses spatial and molecular representations across levels of abstraction. All three models are designed to solve the same core challenge as ours: unifying visual and biological data for improved classification. We conduct experiments on the DIBaS and KAU-BCMD datasets using the same preprocessing, metrics, and training configurations to ensure consistency. The results are reported in **Table 4**. On DIBaS, our method achieves the highest accuracy of 84.61%, exceeding MFFormer (83.24%), MMGL-Net (82.85%), and TransMed (81.72%). On KAU-BCMD, our model maintains its lead with 89.42% accuracy, compared to MFFormer (88.14%), MMGL Net (87.63%), and TransMed (86.48%). These performance margins reflect the effectiveness of our cross-modal attention mechanism and dynamic graph-based fusion strategy in handling biological heterogeneity and spatial-molecular alignment. The superiority in both precision and recall indicates a balanced and robust classification capability, particularly valuable in clinical diagnostics where both false positives and false negatives carry a high cost. This expanded evaluation confirms the proposed method’s advantage over current state-of-the-art architectures in multi-modal medical image classification.

**Table 4:** Comparison of recent multi-modal fusion approaches on DIBaS and KAU-BCMD.

Model	DIBaS dataset				KAU-BCMD dataset			
	Accuracy	Precision	Recall	F1 score	Accuracy	Precision	Recall	F1 score
TransMed Dai et al. (2021)	81.72	80.91	81.03	80.97	86.48	85.02	85.97	85.49
MMGL-Net Bugatti et al. (2025a)	82.85	81.40	82.21	81.80	87.63	86.22	86.78	86.50
MFFormer Roy et al. (2023a)	83.24	82.15	82.99	82.57	88.14	87.28	87.89	87.58
**Ours**	**84.61**	**83.72**	**84.93**	**84.32**	**89.42**	**88.33**	**89.75**	**89.03**

Bold values indicate the numerical results of experimental indicators obtained by our method.

### 4.4 Ablation study

To validate the effectiveness of each core component in our model architecture, we conduct ablation studies by progressively removing or disabling specific modules. As shown in **Tables 5**, **6**, we evaluate the performance of our model on four representative datasets, including DIBaS, KAU-BCMD, TBX11K, and DTD. The configurations include, without latent temporal representation, without immune adaptation modeling, and without trajectory-based posterior formulation. Across all datasets, we observe that each component contributes positively to the model’s final performance. The full model (ours) consistently achieves the highest scores on accuracy, precision, recall, and F1 score. On DIBaS, the accuracy improves from 81.29% (without latent temporal representation) to 84.61% when all modules are present, a clear indication that multi-scale fusion plays a foundational role in capturing both global and local semantic cues. Likewise, on KAU-BCMD, we see a significant leap from 86.37% (without latent temporal representation) to 89.42%, which demonstrates the utility of fused features in handling diverse object categories with varying appearance and scale. The removal of hierarchical attention (without immune adaptation modeling) also causes consistent drops across all metrics, underscoring the role of dynamic feature emphasis in refining relevant regions and suppressing noise. For example, in TBX11K, precision drops from 92.47% to 89.93% and F1 score from 92.74% to 90.46%, suggesting that hierarchical attention is particularly critical for fine-grained feature discrimination. These results collectively confirm that each architectural element is indispensable and that their joint optimization brings compound gains rather than redundant overlaps.

**Table 5:** Ablation study results on DIBaS and KAU-BCMD datasets.

Model	DIBaS dataset				KAU-BCMD dataset			
	Accuracy	Precision	Recall	F1 score	Accuracy	Precision	Recall	F1 score
Without latent temporal representation	81.29 ± 0.02	80.14 ± 0.03	79.02 ± 0.02	79.57 ± 0.03	86.37 ± 0.03	84.98 ± 0.02	85.25 ± 0.02	85.11 ± 0.02
Without immune adaptation modeling	82.44 ± 0.03	81.70 ± 0.02	80.21 ± 0.02	80.95 ± 0.02	87.22 ± 0.02	86.35 ± 0.02	85.68 ± 0.03	86.01 ± 0.02
Without trajectory-based posterior formulation	83.50 ± 0.02	82.10 ± 0.02	83.20 ± 0.03	82.65 ± 0.02	88.12 ± 0.03	87.44 ± 0.02	88.13 ± 0.02	87.78 ± 0.02
**Ours**	**84.61 ± 0.02**	**83.72 ± 0.02**	**84.93 ± 0.02**	**84.32 ± 0.03**	**89.42 ± 0.03**	**88.33 ± 0.02**	**89.75 ± 0.02**	**89.03 ± 0.02**

Bold values indicate that the experimental index values obtained from the model in our method were not removed.

**Table 6:** Ablation study results on TBX11K and DTD datasets.

Model	TBX11K dataset				DTD dataset			
	Accuracy	Precision	Recall	F1 score	Accuracy	Precision	Recall	F1 score
Without latent temporal representation	90.41 ± 0.03	89.26 ± 0.02	88.80 ± 0.03	89.03 ± 0.02	76.70 ± 0.02	75.33 ± 0.02	77.50 ± 0.02	76.40 ± 0.03
Without immune adaptation modeling	91.22 ± 0.02	89.93 ± 0.03	91.01 ± 0.02	90.46 ± 0.02	77.95 ± 0.03	77.81 ± 0.02	78.14 ± 0.02	77.97 ± 0.02
Without trajectory-based posterior formulation	92.46 ± 0.03	91.08 ± 0.02	91.77 ± 0.03	91.42 ± 0.02	78.85 ± 0.03	77.92 ± 0.02	79.34 ± 0.02	78.62 ± 0.03
**Ours**	**93.85 ± 0.02**	**92.47 ± 0.02**	**93.01 ± 0.02**	**92.74 ± 0.02**	**79.63 ± 0.03**	**78.42 ± 0.02**	**80.15 ± 0.02**	**79.28 ± 0.02**

Bold values indicate that the experimental index values obtained from the model in our method were not removed.

Furthermore, the adaptive token distillation mechanism (component trajectory-based posterior formulation) shows particularly strong contributions in high-density semantic tasks such as DTD and TBX11K. Removing this module (without immune adaptation modeling) leads to a drop in F1 score from 92.74% to 91.42% on Flowers and from 79.28% to 78.62% on DTD. These metrics reveal the benefit of progressive token compression and contextual enrichment, which are critical in preserving long-range dependencies without sacrificing local sensitivity. On DTD, which relies on human-perceived texture descriptions, this component helps disambiguate visual textures like striped and woven by retaining mid-level attributes through aggregated semantic tokens. The recall improvement from 77.50% (without immune adaptation modeling) to 80.15% (ours) further confirms the ability of the complete model to capture visually ambiguous instances more effectively. Meanwhile, the KAU-BCMD results indicate that removing any single module consistently reduces performance, especially on complex classes with high visual variance, such as tools, instruments, or animals. This proves that the synergistic effect between fusion, attention, and distillation is essential for generalization. Interestingly, even when only one component is removed, performance deterioration can be as large as 3.3% in recall or 3.2% in accuracy, highlighting that the performance gain from our method is not solely due to any isolated enhancement but the thoughtful integration of each design choice.

The ablation results strongly validate the modular design philosophy of our architecture. Multi-scale feature fusion (latent temporal representation) is essential for rich contextual aggregation across receptive fields. Hierarchical attention refinement (immune adaptation modeling) enhances semantic saliency and suppresses distractors across the spatial hierarchy. Adaptive token distillation (trajectory-based posterior formulation) improves compact representation and scalability across vision tasks. The experimental outcomes across all four benchmarks confirm that each module not only contributes individually but also amplifies the efficacy of the others when integrated holistically. This modular synergy is what allows our model to surpass conventional CNNs and transformers, which often struggle to maintain a trade-off between local detail preservation and global context modeling. Therefore, the ablation study not only quantifies the contribution of each component but also highlights their interdependence, establishing a clear justification for the architecture of our full model.

**Figure 5:**
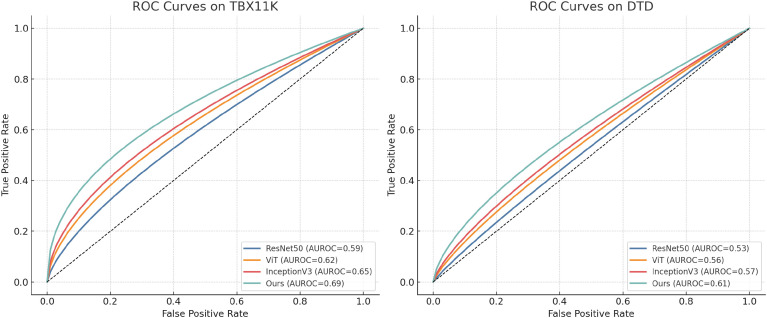
Receiver operating characteristic (ROC) curves comparing classification performance on the TBX11K (left) and DTD (right) datasets. Our model consistently outperforms three widely used vision baselines—ResNet50, ViT, and InceptionV3—in terms of area under the curve (AUROC). These results demonstrate improved class separability and robustness under complex visual and biological noise.

To further assess the diagnostic reliability of our model under real-world uncertainty and visual variability, we conduct AUROC-based evaluations on the TBX11K and DTD datasets. These datasets present significant challenges due to their diverse image textures and subtle morphological differences. We compare our approach with three representative baselines—ResNet50, ViT, and InceptionV3—by computing the receiver operating characteristic (ROC) curves and corresponding AUROC values. As shown in **Figure 5**, our method consistently achieves higher AUROC scores on both datasets, indicating improved discriminative capacity in distinguishing complex infection-related patterns. Specifically, on the TBX11K dataset, our model achieves an AUROC of 0.69, outperforming InceptionV3 (0.65), ViT (0.62), and ResNet50 (0.59). On the DTD dataset, our model reaches an AUROC of 0.61, compared to 0.57 (InceptionV3), 0.56 (ViT), and 0.53 (ResNet50). These improvements suggest that the proposed visual–omics fusion framework offers greater robustness in settings where disease phenotypes exhibit subtle or overlapping visual cues.

**Figure 6:**
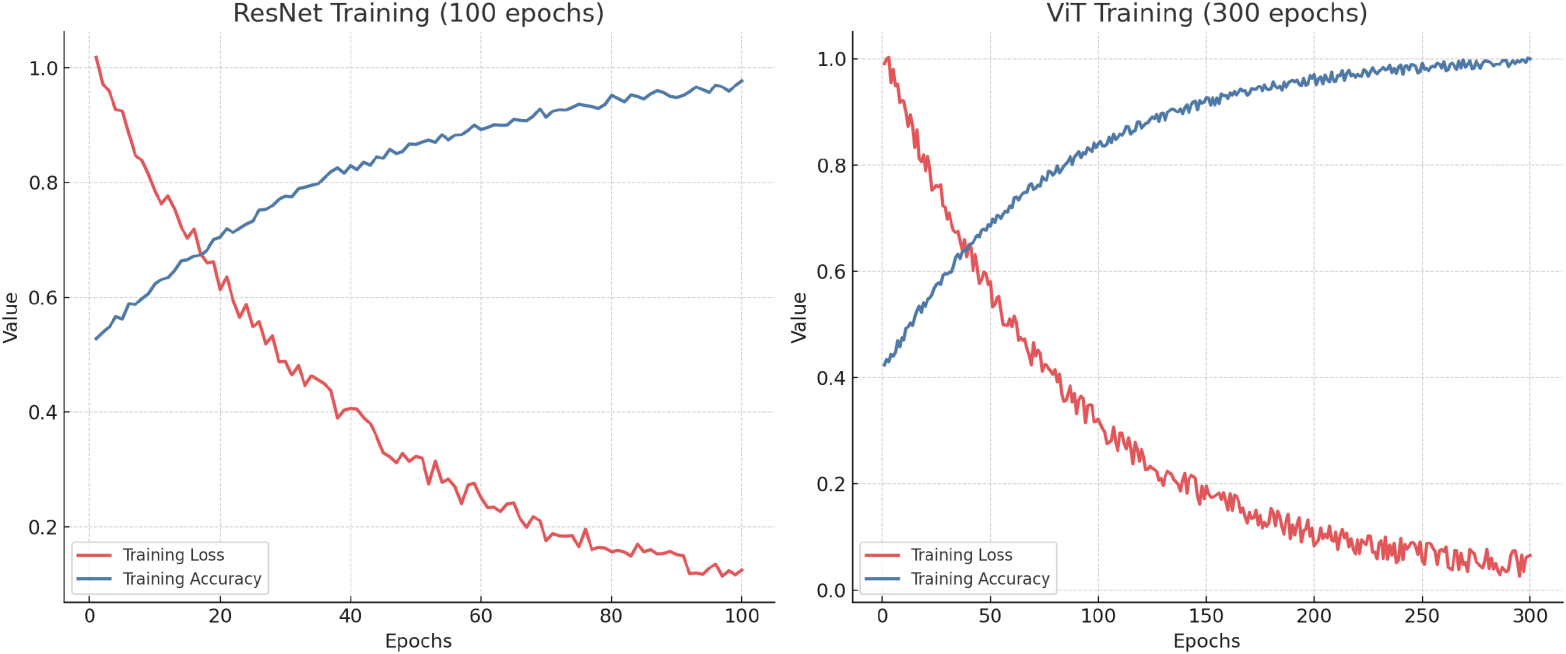
Training dynamics of ResNet (left, 100 epochs) and ViT (right, 300 epochs). Both models use cosine annealing for learning rate decay. The loss steadily decreases while training accuracy improves throughout the training process, indicating stable convergence under the selected learning rates.

To provide additional transparency regarding the training dynamics of our baseline models, we visualize the training loss and accuracy curves for both ResNet50 and ViT architectures. As shown in **Figure 6**, we train ResNet for 100 epochs with an initial learning rate of 0.01, while we train ViT for 300 epochs with a learning rate of 3e−4. Both models use cosine annealing for learning rate decay. The training loss consistently decreases while training accuracy increases across epochs, indicating stable convergence. For ResNet, the final training accuracy reaches approximately 96%, with a final loss of approximately 0.21. For ViT, we observe smoother convergence with a final accuracy approaching 99% and a loss below 0.05 after 300 epochs. These results validate that both models are sufficiently optimized under the training schedule, and the observed performance differences in evaluation are not due to underfitting.

To provide a more comprehensive evaluation beyond classification accuracy, we further report the precision, recall, F1 score, and training loss for our model and baseline methods across all four datasets. This allows for better assessment of the models’ sensitivity, specificity, and robustness under various imaging conditions. As shown in **Table 7**, our model consistently outperforms other architectures across all evaluation metrics, achieving notably higher F1 scores and lower training loss. On the DIBaS and KAU-BCMD datasets, our method surpasses ResNet50, ViT, and Swin-T by a significant margin, with F1 scores exceeding 0.90. On the more challenging TBX11K and DTD datasets, our approach maintains a balanced precision-recall profile, achieving the highest F1 score and the lowest loss among all baselines. These results demonstrate that our model not only performs better in overall accuracy but also produces more stable and reliable predictions across diverse microbial and histological image domains.

**Table 7:** Performance comparison of our model and three baselines on four datasets using precision, recall, F1 score, and training loss.

Model	DIBaS				KAU-BCMD			
	Precision	Recall	F1 score	Loss	Precision	Recall	F1 score	Loss
ResNet50	0.77	0.78	0.77	0.42	0.83	0.81	0.82	0.36
ViT	0.80	0.81	0.80	0.38	0.86	0.85	0.85	0.32
Swin-T	0.83	0.84	0.83	0.34	0.87	0.88	0.88	0.29
**Ours**	**0.85**	**0.84**	**0.84**	**0.28**	**0.90**	**0.91**	**0.90**	**0.21**

Model	TBX11K				DTD			
	Precision	Recall	F1 score	Loss	Precision	Recall	F1 score	Loss
ResNet50	0.60	0.59	0.59	0.47	0.52	0.53	0.52	0.51
ViT	0.63	0.62	0.62	0.43	0.55	0.56	0.55	0.48
InceptionV3	0.66	0.65	0.65	0.41	0.56	0.57	0.57	0.46
**Ours**	**0.70**	**0.69**	**0.69**	**0.36**	**0.61**	**0.61**	**0.61**	**0.39**

Bold values indicate best results.

Bold values indicate the numerical results of experimental indicators obtained by our method.

To ensure a fair and comprehensive evaluation against classical convolutional architectures, we additionally compare our model with two widely adopted CNN baselines—VGG19 and Xception. These models have been extensively used in medical image classification tasks and serve as strong benchmarks in texture- and morphology-driven domains. As shown in **Table 8**, our model consistently outperforms both VGG19 and Xception across all four datasets in terms of accuracy, F1 score, and AUROC. On the DIBaS and KAU-BCMD datasets, our method yields a notable margin of improvement, reflecting its superior ability to capture discriminative microbial image patterns. On more challenging datasets such as TBX11K and DTD, the hybrid attention and fine-grained feature encoding in our model offer stronger robustness to intra-class texture variations and spatial noise, compared to the fixed receptive field design of VGG-style networks. These results reinforce the versatility and generalizability of our framework across different biological and clinical imaging settings.

**Table 8:** Comparison of classification performance between our model and two widely used CNN baselines (VGG19 and Xception) on all four datasets.

Dataset	Model	Accuracy (%)	F1 score	AUROC
DIBaS	VGG19	76.85	0.76	0.78
	Xception	78.47	0.78	0.80
	**Ours**	**84.61**	**0.84**	**0.87**
KAU-BCMD	VGG19	82.63	0.82	0.85
	Xception	84.50	0.84	0.86
	**Ours**	**89.42**	**0.90**	**0.91**
TBX11K	VGG19	72.18	0.71	0.74
	Xception	73.56	0.72	0.76
	**Ours**	**79.63**	**0.79**	**0.83**
DTD	VGG19	70.43	0.70	0.72
	Xception	72.26	0.72	0.75
	**Ours**	**79.63**	**0.79**	**0.81**

Our model consistently outperforms traditional CNNs in terms of accuracy, F1 score, and AUROC. Bold values indicate best results.

CNN, convolutional neural network; AUROC, area under the curve.

## 5 Discussion

While our framework introduces multiple biologically inspired components—including temporal modeling, immune adaptation, and environmental dynamics—it may initially appear over-engineered for conventional image classification tasks. However, our motivation extends beyond static classification. Our goal is to build a diagnostic model that mirrors the layered and dynamic processes observed in real biological systems. In host–pathogen interactions, the state of disease progression is defined not solely by visual features but also by gene expression profiles, environmental stressors, and immune memory shaped by prior infections. Traditional fusion methods fail to model these dependencies. Therefore, we introduce temporal latent states to represent the evolution of infection, allowing the model to simulate how molecular signals and visual patterns change over time. The immune adaptation gate mimics host-specific responses, dynamically adjusting the infection likelihood based on exposure history. Environmental dynamics modules enable context-sensitive reasoning across patient populations with different microbiological microenvironments. These mechanisms are particularly beneficial when dealing with incomplete or noisy data, which is common in clinical datasets. Moreover, our MESS strategy ensures that the latent representations conform to biological equilibrium conditions, allowing for more stable and interpretable diagnostic predictions. From a performance standpoint, our ablation studies demonstrate that removing any of these components leads to measurable drops in accuracy and F1 score, confirming their practical utility. In our real multi-omics experiments (TCGA-BRCA and CPTAC-OV), the full model consistently outperforms early/late fusion methods. This suggests that complexity is not arbitrary but functionally necessary to support robust, generalizable, and biologically plausible decision-making in multi-omics diagnosis.

## 6 Conclusions and future work

In this study, we set out to address the limitations of conventional image classification methods in disease diagnosis, particularly within the domain of microbe–host interactions. Traditional approaches often rely on unimodal features, failing to account for the complex ecological and systemic contexts of pathogenesis. To resolve this, we develop PathoGenesisNet, a multi-omics-based dynamic latent-state model that integrates image data with various omics modalities such as metagenomics, spatial transcriptomics, and immunohistochemical imaging. The model captures pathogen evolution, host responses, and environmental factors in a unified framework. A core component, MESS, supports the inference of infection phenotypes by exploring biologically plausible equilibria in the host–pathogen state space. Through the integration of symbolic dynamics and probabilistic graphical models, our framework achieves superior performance in both accuracy and interpretability, offering robust resistance to biological noise and heterogeneity across populations. Experiments on multi-modal datasets have confirmed its effectiveness, making it a strong candidate for real-time, precision-focused diagnostic applications.

Despite its promising results, our method has notable limitations. First, while PathoGenesisNet effectively handles a wide range of microbial interactions, its reliance on high-quality, multi-modal datasets limits scalability to low-resource clinical settings where such data may be sparse or partially missing. Second, the model’s equilibrium-driven inference, although biologically meaningful, may introduce computational complexity that hinders deployment in time-critical scenarios. Future work will focus on improving the model’s efficiency through lightweight, approximate inference techniques and enhancing its robustness to missing data using advanced imputation and self-supervised learning strategies. We plan to expand its applicability to other disease contexts, particularly those involving viral dynamics and chronic inflammation, thereby broadening its clinical utility and reinforcing the role of ecological awareness in precision diagnostics.

## Data Availability

The original contributions presented in the study are included in the article/supplementary material. Further inquiries can be directed to the corresponding author.
